# Dataset of *de novo* assembly and functional annotation of the transcriptome of blueberry (*Vaccinium* spp.)

**DOI:** 10.1016/j.dib.2019.104390

**Published:** 2019-08-12

**Authors:** Xinpeng Qi, Elizabeth L. Ogden, Mark K. Ehlenfeldt, Lisa J. Rowland

**Affiliations:** a USDA-ARS, BARC-West, Genetic Improvement of Fruits and Vegetables Laboratory, Beltsville, MD 20705, USA; b USDA-ARS, Genetic Improvement of Fruits and Vegetables Laboratory, at Rutgers University, P.E. Marucci Center for Blueberry and Cranberry Research and Extension, Chatsworth, NJ 08019, USA

**Keywords:** Blueberry, Transcriptome assembly, RNA-Seq, *Vaccinium* spp.

## Abstract

Blueberry is an economically important berry crop. Both production and consumption of blueberries have increased sharply worldwide in recent years at least partly due to their known health benefits. The development of improved genomic resources for blueberry, such as a well-assembled genome and transcriptome, could accelerate breeding through genomic-assisted approaches. To enrich available transcriptome data and identify genes potentially involved in fruit quality, RNA sequencing was performed on fruit tissue from two northern-adapted hybrid blueberry breeding populations. RNA-seq was carried out using the Illumina HiSeq^TM^ 2500 platform. Because of the absence of a reference-grade genome for blueberry, a transcriptome was *de novo* assembled from this RNA-seq data and other publicly available transcriptome data from blueberry downloaded from the National Center for Biotechnology Information (NCBI) Short Read Archive (SRA) using Trinity. After removing redundancy, this resulted in a dataset of 91,861 blueberry unigenes. This unigene dataset was functionally annotated using the NCBI-Nr protein database. All raw reads from the breeding populations were deposited in the NCBI SRA with accession numbers SRR6281886, SRR6281887, SRR6281888, and SRR6281889. The *de novo* transcriptome assembly was deposited at NCBI Transcriptome Shotgun Assembly (TSA) database with accession number GGAB00000000. These data will provide real expression evidence for the blueberry genome gene prediction and gene functional annotation and a reference transcriptome for future gene expression studies involving blueberry fruit.

**Table:** Specifications Table

Subject area	*Biology*
More specific subject area	*Transcriptomics*
Type of data	*Transcriptome sequences*
How data was acquired	*Illumina HiSeq^TM^ 2500 sequencing platform*
Data format	*Raw and analyzed*
Experimental factors	*Ripe fruit was collected from two field-grown blueberry breeding populations segregating for the waxy coating on fruit*
Experimental features	*Total RNA was extracted from bulked waxy and non-waxy fruit tissue from two blueberry breeding populations segregating for the waxy coating on fruit. RNA was used for cDNA library construction. Paired-end RNA-seq data was generated using the Illumina HiSeq^TM^ 2500 sequencing platform. Trinity software was used to perform de novo assembly of clean reads from this data and other blueberry transcriptome data downloaded from NCBI SRA. The unigene dataset was functionally annotated using NCBI-Nr protein database.*
Data source location	*USDA-ARS, Genetic Improvement of Fruits and Vegetables Laboratory, Beltsville, MD 20705, USA*
Data accessibility	*NCBI SRA accession numbers of SRR6281886(**https://www.ncbi.nlm.nih.gov/sra/SRR6281886**)*, *SRR6281887 (**https://www.ncbi.nlm.nih.gov/sra/SRR6281887**)*, *SRR6281888 (**https://www.ncbi.nlm.nih.gov/sra/SRR6281888**)*, *and SRR6281889 (**https://www.ncbi.nlm.nih.gov/sra/SRR6281889**)**for raw reads;* *NCBI TSA accession number of GGAB00000000 (**https://www.ncbi.nlm.nih.gov/nuccore/GGAB00000000.1/**)**for assembly*
Related research article	*Xinpeng Qi, Elizabeth L. Ogden, Mark K. Ehlenfeldt, and Lisa J. Rowland. Transcriptome analysis identifies genes related to the waxy coating on blueberry fruit in two northern-adapted rabbiteye breeding populations, “under review”.*

**Table:** 

**Value of the Data** 1. The RNA-seq and assembled transcriptome datasets provide real expression evidence for use to the scientific research community in gene prediction and functional annotation of the emerging blueberry genome. 2. The RNA-seq dataset is useful to scientists in identifying differentially expressed genes between blueberry fruit with and without the waxy coating and, thus, genes associated with wax accumulation in berry fruit. 3. The expression profiles are available as raw sequence reads that can be further processed and analyzed by researchers using their own bioinformatic algorithms. 4. The *de novo* assembled transcriptome is useful as a reference transcriptome to other scientists working to identify differentially expressed genes in blueberry and in related *Vaccinium* species.

## 1 Data

**Fig. 1:**
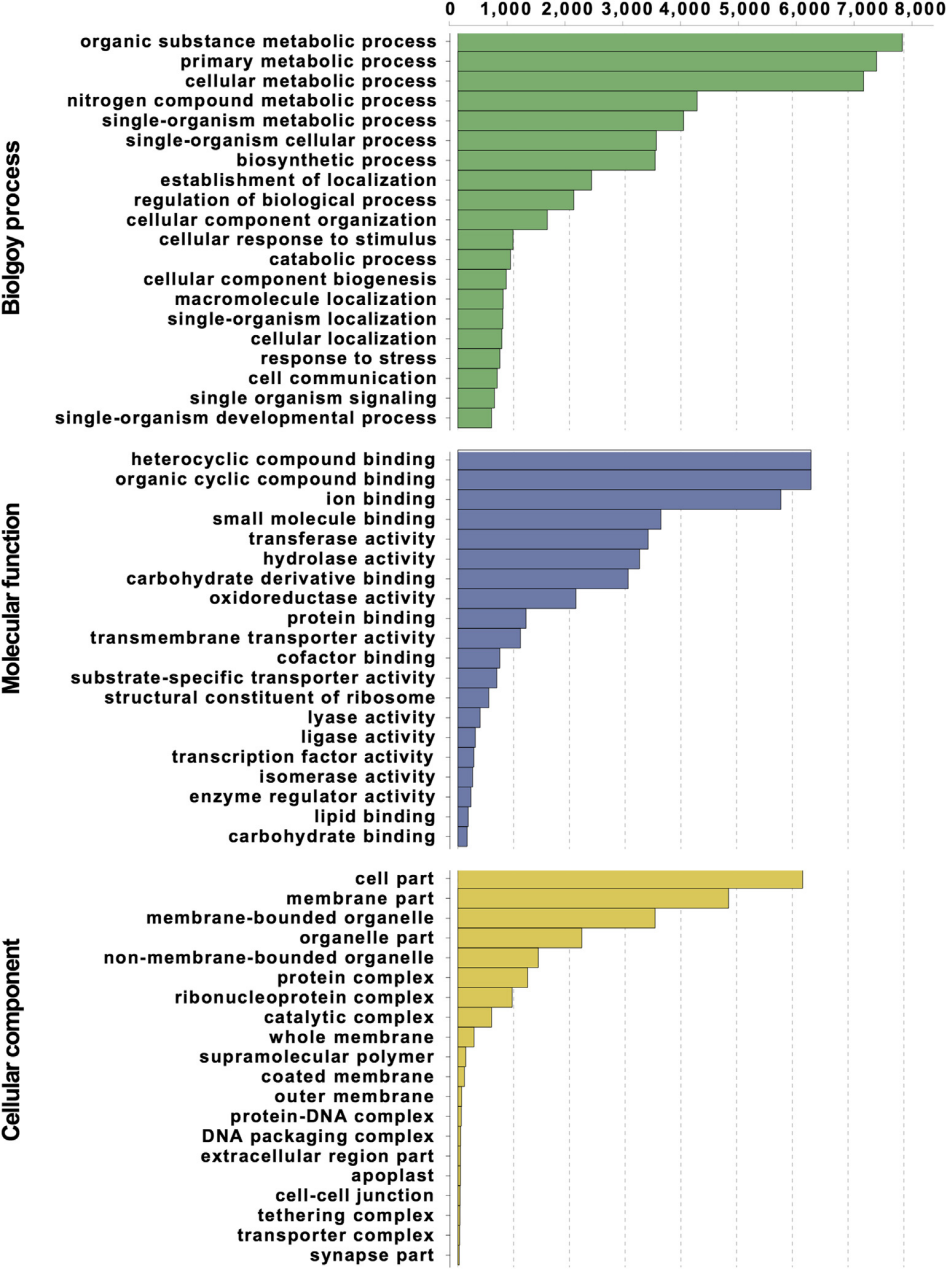
GO category distribution of Blast2GO annotated blueberry unigenes.

The transcriptomes of two northern-adapted rabbiteye hybrid blueberry breeding populations were generated from RNA extracts of bulked fruit tissue samples of plants with and without the epicuticular waxy coating on the fruit. The four cDNA libraries from the waxy and non-waxy bulks of the two populations were sequenced via the Illumina HiSeq^TM^ 2500 platform resulting in approximately 317 million RNA-seq reads (Table 1). The raw reads were subjected to quality control measures, and this dataset, along with other publicly available RNA-seq data from blueberry, were assembled (Table 2) as described in the following sections. The other publicly available RNA-seq datasets included: SRR1187632, SRR1187673, SRR1187674, SRR1187675, SRR1187676, SRR1187677, SRR1188088, SRR1188089, SRR1188090, SRR1188091, SRR1188222, SRR1188230, SRR1188236, SRR1188240, SRR1188242, SRR1188247, SRR1188258, SRR1188265, SRR1188270, SRR1188282, SRR1188283, SRR942391, and SRR950441. After removing redundancy, this assembly was functionally annotated by NCBI-Nr protein database and then assigned Gene Ontology (GO) identity (Fig. 1). Both raw RNA-seq reads and the *de novo* transcriptome assembly can be accessed in the NCBI repository, with the SRA accession number SRR6281886 for ‘Nocturne’ x T 300 population non-waxy library, SRR6281887 for ‘Nocturne’ x T 300 population waxy library, SRR6281888 for ‘Nocturne’ x US 1212 population non-waxy library, and SRR6281889 for ‘Nocturne’ x US 1212 population waxy library, and with the TSA accession number GGAB00000000 for the assembled unigene transcriptome dataset. The expression profiles revealed from analysis of the RNA-seq data and the resulting *de novo* transcriptome assembly will help with gene identification and annotation of the emerging blueberry genome.

**Table 1:** RNA-seq of two northern-adapted rabbiteye hybrid blueberry breeding populations.

Library	Number of plants in bulk	Number of clean reads
‘Nocturne’ x T 300 non-waxy	9	69,318,222
‘Nocturne’ x T 300 waxy	10	97,629,788
‘Nocturne’ x US 1212 non-waxy	10	68,222,558
‘Nocturne’ x US 1212 waxy	13	81,404,420

**Table 2:** Statistics of the blueberry transcriptome raw assembly, quality assessment, and redundancy elimination.

Total “genes” from Trinity assembly	251,974
Assembly N50 length	806
Total assembled bases	208,124,831
‘Nocturne’ x T 300 non-waxy (%)	87.16^a^
‘Nocturne’ x T 300 waxy (%)	92.00^a^
‘Nocturne’ x US 1212 non-waxy (%)	93.36^a^
‘Nocturne’ x US 1212 waxy (%)	91.63^a^
Total full-length SwissProt protein hits	9909
Total unigene number	91,861
Unigene N50 length	1144
Total unigene bases	66,642,205

a Mapping rate of mapping RNA-seq reads back to the *de novo* assembly.

## 2 Experimental design, materials, and methods

### 2.1 Plant materials and sequencing

The two northern-adapted rabbiteye (*Vaccinium virgatum*) hybrid breeding populations resulted from crosses of the cultivar ‘Nocturne’ (dark-fruited with no visible waxy coating) [1] with the selection T 300 (light-blue colored fruit with waxy coating) and the selection US 1212 (light-blue colored fruit with waxy coating). Both populations were grown in field plots at the Blueberry and Cranberry Research Station of Rutgers University, in Chatsworth, NJ, USA, and both segregated visibly for the presence/absence of the waxy coating on the fruit. RNA was extracted [2] from bulked ripe fruit tissues from waxy and non-waxy plants (∼10 plants of each type) from the two populations (Table 1). Complementary DNA libraries for RNA-seq were prepared and sequenced at the David H. Murdock Research Institute in Kannapolis, NC, USA.

### 2.2 Data analysis (raw reads handling and *de novo* assembly)

Raw reads were subjected to quality control by first discarding 10 bp and 5 bp from the 5′ and 3' ends of the 100 bp paired-end reads, and then filtering out reads with >10 Ns out of the remaining 85 bp. Raw and clean reads were checked for quality using FastQC [3]. A laddered *de novo* assembly was performed using Trinity [4] based on clean reads from this study and other blueberry transcriptome data downloaded from NCBI SRA depository under accession numbers SRP039977 (SRR1188088, SRR1188089, SRR1188090, SRR1188091, SRR1188222, SRR1188230, SRR1188236, SRR1188240, SRR1188242, SRR1188247, SRR1188258, SRR1188265, SRR1188270, SRR1188282, SRR1188283), SRP039971 (SRR1187632, SRR1187673, SRR1187674, SRR1187675, SRR1187676, SRR1187677), and SRA046311 (SRR942391, SRR950441). A total of 251,974 raw assembled “genes” were obtained. Two independent methods, mapping clean reads back to the assembly using Bowtie [5], and identifying full-length protein hits using BLASTN against SwissProt database [6], were applied to confirm the high quality of the assembly (Table 2). The raw assembly was then subjected to CD-HIT [7] and TIGR Gene Indices CLustering tools (TGICL) [8] to eliminate redundancy, resulting in a final 91,861 unigene dataset. This non-redundant unigene dataset was functionally annotated by NCBI-Nr protein database using BLASTP, resulting in 56,175 unigenes with high quality hits (identity score no less than 40; hit score no less than 60; hit length no less than half of query protein sequence length). Each unigene of the annotated record was then attempted assignment of GO identity using Blast2GO [9] (GO weight = 5; E-value hit filter = 1e-6), resulting in 19,110 unigenes with assigned GO identities.

## Funding

This project was funded by USDA ARS [Project 8042-21000-279-00].
